# Supplementary material for: ATase inhibition rescues age-associated proteotoxicity of the secretory pathway
Source: Commun Biol. 2022 Feb 25;5:173. doi: 10.1038/s42003-022-03118-0 (PMC8881600; doi:10.1038/s42003-022-03118-0)
Supplement: Supplementary file 2 — Supplementary Information [file 42003_2022_3118_MOESM2_ESM.pdf]

SUPPLEMENTARY INFORMATION

ATase inhibition rescues age-associated proteotoxicity of the secretory pathway

Maeghan Murie, Yajing Peng, Michael J. Rigby, Inca A. Dieterich, Mark A. Farrugia,

Andreas Endresen, Anita Bhattacharyya, Luigi Puglielli

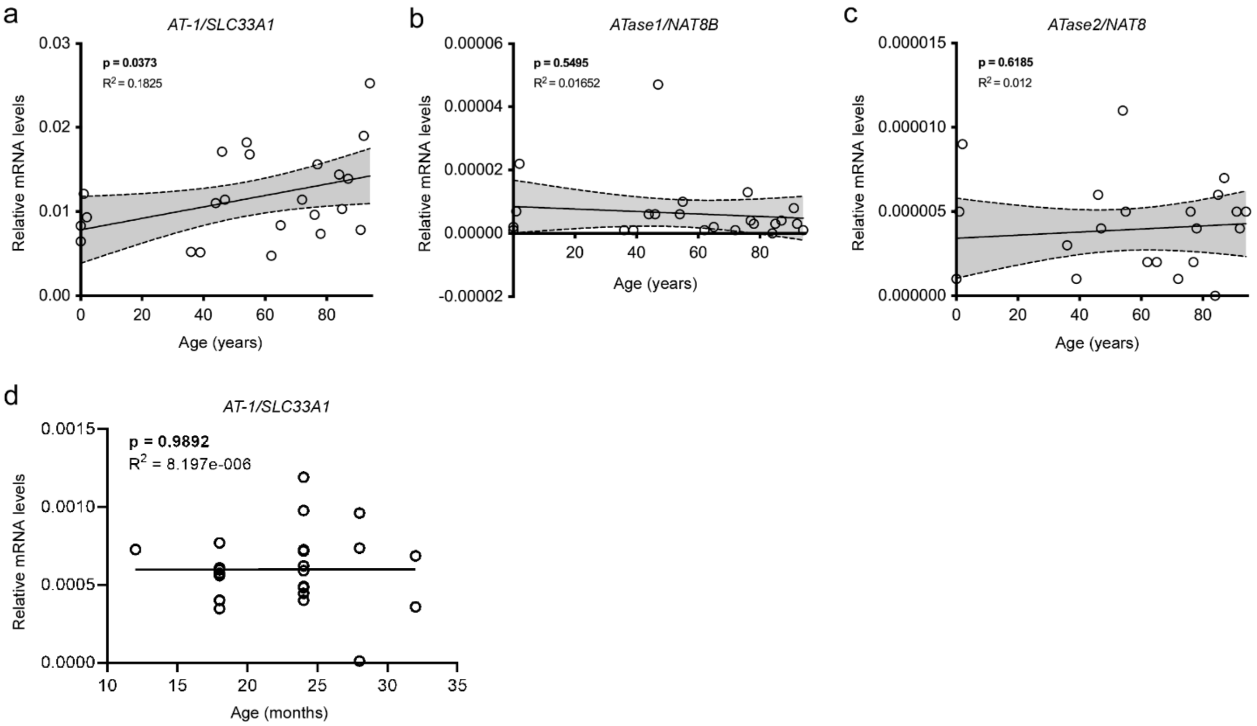

Supplementary Figure 1. ***AT-1*** **is upregulated as a function of age in human fibroblasts.** (**a-c**) mRNA levels of *AT-1* were measured in 24 human fibroblasts, and normalized to *GAPDH* expression. A univariate linear regression analysis was run (Y = 9.149e-009*X + 3.419e-006; F=0.2554) and significance is reported within the figure. (**d**) mRNA levels of *AT-1* were measured in the brain of C57BL/6 from the NIA aging mouse cohort, and normalized to *GAPDH* levels.

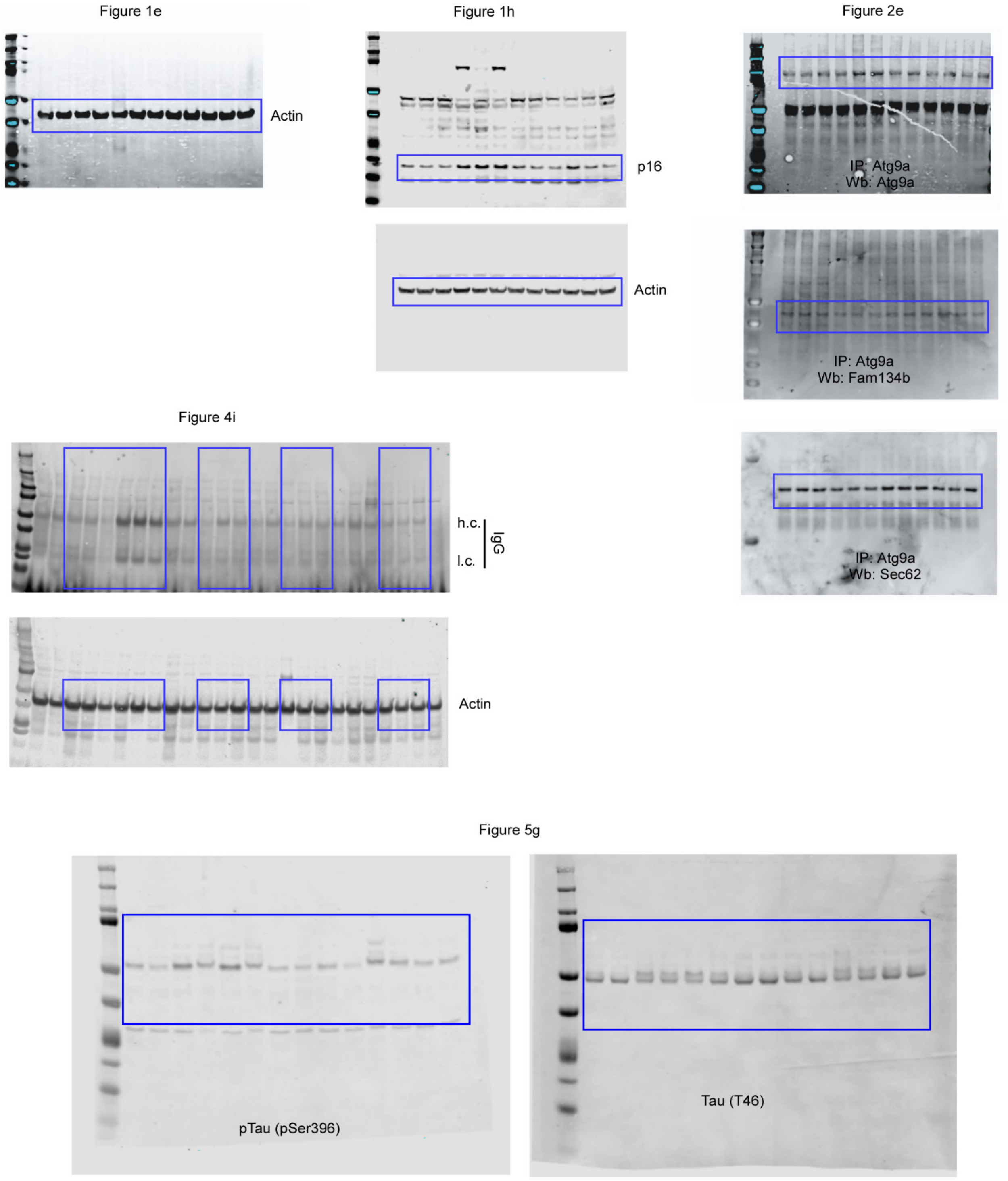

Supplementary Figure 2. Uncropped Gels.

Figure 6b

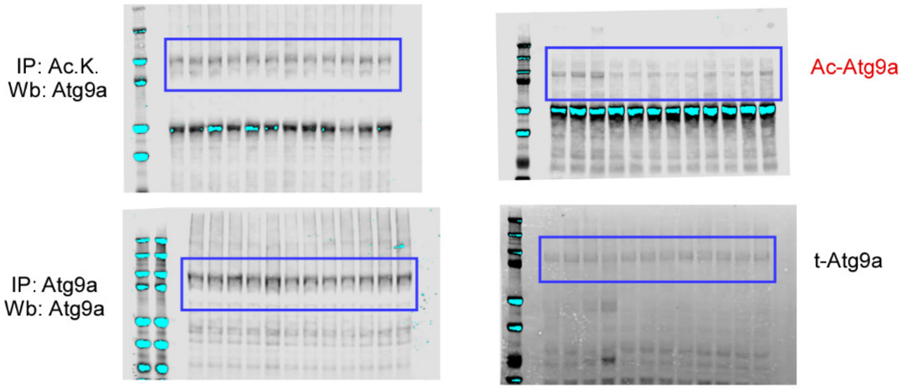

Figure 6e

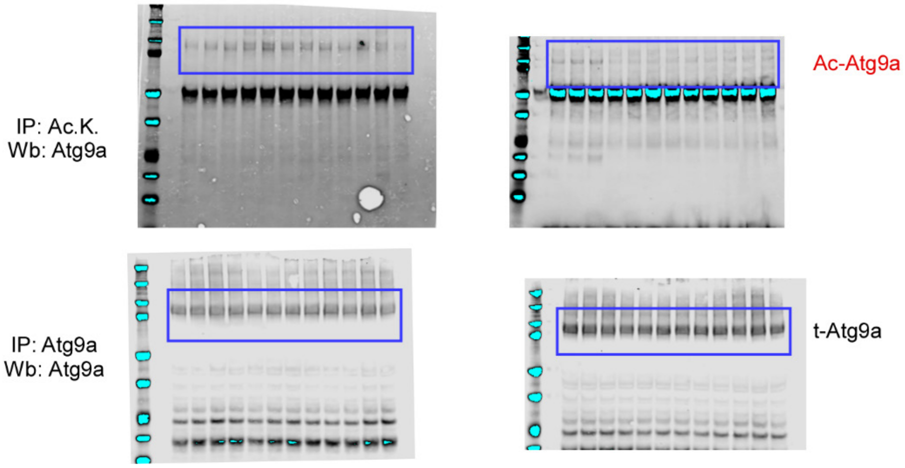

Supplementary Figure 3. Uncropped Gels.

Supplementary Table 1

List of the ATase inhibitors shown in Figure 3b and 3d

**Compound**	**ZINC15 ID**	**ATase 1** (% inhibition)	**ATase 2** (% inhibition)
Cmp 1	166974	53	63
Cmp 2	1038971	53	83
Cmp 3	3128876	40	78
Cmp 4	95742794	57	86
Cmp 5	116271	55	85
Cmp 6	2573087	49	65
Cmp 7	3957707	49	80
Cmp 8	1036814	56	90
Cmp 9	4037676	74	88
Cmp10	155800	52	80
Cmp 11	160333	43	74
Cmp 12	163101	42	84
Cmp 13	171969	37	78
Cmp 14	172750	50	76
Cmp 15	71157	52	78
Cmp 16	71274	52	68
Cmp 17	4381858	66	85
Cmp 18	13467976	25	65
Cmp 19	87491	56	74
Cmp 20	87596	56	83
Cmp 21	4361452	60	64
Cmp 22	97832	50	76
Cmp 23	4377447	54	78
Cmp 24	102932	50	65
Cmp 25	17284974	50	50
Cmp 26	4390342	55	61
Cmp 27	111475	67	66
Cmp 28	13658476	48	79
Cmp 29	120763	32	81
Cmp 30	1047729	53	81

NOTE: Inhibition is as determined by the high throughput screen. Each compound was used at the final concentration of 10µm. The percent of inhibition is expressed as the mean of N=3.

Supplementary Table 2

Plasma pharmacokinetic parameters of Compound 9 and 19

Parameters	Unit	Compound 9	Compound 19
$T_{1/2}$	h	3.38	10.3
T_{max}	h	2	0.0833
C_{max}	ng/mL	395	235
AUC_{last}	h*ng/mL	2298	297
AUC_{Inf}	h*ng/mL	2903	586
$AUC_{\%Extrap}$ obs	%	20.8	49.4
MRT_{Inf} obs	h	5.41	12.6
AUC_{last}/D	h*mg/mL	230	29.7
F	%	117	807

NOTE: Compounds were administered orally at the final concentration of 10mg/Kg. PK data were collected in C57BL/6 WT/Non-Tg mice (N=3).

Supplementary Table 3.

List of human fibroblasts used with age

Cell line	Age	Source
WC 5803	Neonate	UW-Madison
WC6007	Neonate	UW-Madison
AG01521	3 day	Coriell
AG08498	1 yr	Coriell
AG07095	2 yr	Coriell
WC0603	36 yr	UW-Madison
WC0504	39 yr	UW-Madison
AG06282	44 yr	Coriell
AG12954	46 yr	Coriell
AG07118	47 yr	Coriell
AG13093	54 yr	Coriell
AG05840	55 yr	Coriell
WC0306	62 yr	UW-Madison
AG04357	65 yr	Coriell
WC0405	72 yr	UW-Madison
AG11743	76 yr	Coriell
AG09879	77 yr	Coriell
AG12997	78 yr	Coriell
AG11725	84 yr	Coriell
AG13077	85 yr	Coriell
AG10884	87 yr	Coriell
AG07725	91 yr	Coriell
AG09602	92 yr	Coriell
AG08433	94 yr	Coriell
