# Supplementary material for: ATase inhibition rescues age-associated proteotoxicity of the secretory pathway
Source: Commun Biol. 2022 Feb 25;5:173. doi: 10.1038/s42003-022-03118-0 (PMC8881600; doi:10.1038/s42003-022-03118-0)
Supplement: Supplementary file 3 — Description of Additional Supplementary Files [file 42003_2022_3118_MOESM3_ESM.pdf]

Description of Additional Supplementary Files

File name: Supplementary Data 1
Description: List of compounds analyzed with the Modified Lipinski's Rule (for CNS delivery).

File name: Supplementary Data 2
Description: Data underlying the graphs and charts within the main figures.
