# Supplementary material for: ATase inhibition rescues age-associated proteotoxicity of the secretory pathway
Source: Commun Biol. 2022 Feb 25;5:173. doi: 10.1038/s42003-022-03118-0 (PMC8881600; doi:10.1038/s42003-022-03118-0)
Supplement: Supplementary file 6 — Reporting Summary [file 42003_2022_3118_MOESM6_ESM.pdf]

nature research

Corresponding author(s): Luigi Puglielli

Last updated by author(s): Jan 24, 2022

Reporting Summary

Nature Research wishes to improve the reproducibility of the work that we publish. This form provides structure for consistency and transparency in reporting. For further information on Nature Research policies, see our Editorial Policies and the Editorial Policy Checklist.

Statistics

For all statistical analyses, confirm that the following items are present in the figure legend, table legend, main text, or Methods section.

n/a	Confirmed	
☐	☒	The exact sample size (n) for each experimental group/condition, given as a discrete number and unit of measurement
☐	☒	A statement on whether measurements were taken from distinct samples or whether the same sample was measured repeatedly
☐	☒	The statistical test(s) used AND whether they are one- or two-sided<br>*Only common tests should be described solely by name; describe more complex techniques in the Methods section.*
☐	☒	A description of all covariates tested
☒	☐	A description of any assumptions or corrections, such as tests of normality and adjustment for multiple comparisons
☐	☒	A full description of the statistical parameters including central tendency (e.g. means) or other basic estimates (e.g. regression coefficient) AND variation (e.g. standard deviation) or associated estimates of uncertainty (e.g. confidence intervals)
☐	☒	For null hypothesis testing, the test statistic (e.g. F, t, r) with confidence intervals, effect sizes, degrees of freedom and P value noted<br>*Give P values as exact values whenever suitable.*
☒	☐	For Bayesian analysis, information on the choice of priors and Markov chain Monte Carlo settings
☒	☐	For hierarchical and complex designs, identification of the appropriate level for tests and full reporting of outcomes
☒	☐	Estimates of effect sizes (e.g. Cohen's d, Pearson's r), indicating how they were calculated

Our web collection on statistics for biologists contains articles on many of the points above.

Software and code

Policy information about availability of computer code

Data collection	Data was collected using NIS-Elements AR, ImageStudio V5.2, Azure cSeries Acquisition Software, ImagePro V6.3, LightCycler 480 Software, VersaMax Acquisition Software, GloMax Acquisition Software, LICOR Odyssey Infrared Imaging System, UltraFocus DXA system (Faxitron), Hewlett Packard Faxitron X-ray system, HemaVet Complete Blood Count (CBC) instrument, Leica DM4000 B microscope, ImageJ version 2.0, and AutoDock Vina version 1.1.2.
Data analysis	Statistical analyses were performed using GraphPad Prism 8.4.3 and Microsoft Excel. Other data analysis was conducted on Imaris (Bitplane) V9.5, Python Molecular Viewer (PMV) version 1.5.6 software, PyMOL version 2.4.1 software, and WinNonlin (PhoenixTM, version 8.2).

For manuscripts utilizing custom algorithms or software that are central to the research but not yet described in published literature, software must be made available to editors and reviewers. We strongly encourage code deposition in a community repository (e.g. GitHub). See the Nature Research guidelines for submitting code & software for further information.

Data

Policy information about availability of data

All manuscripts must include a data availability statement. This statement should provide the following information, where applicable:

- Accession codes, unique identifiers, or web links for publicly available datasets
- A list of figures that have associated raw data
- A description of any restrictions on data availability

The authors declare that all data supporting the findings of this study are available within the paper and its Supplementary Information and Data.

Field-specific reporting

Please select the one below that is the best fit for your research. If you are not sure, read the appropriate sections before making your selection.

☒ Life sciences ☐ Behavioural & social sciences ☐ Ecological, evolutionary & environmental sciences

For a reference copy of the document with all sections, see nature.com/documents/nr-reporting-summary-flat.pdf

Life sciences study design

All studies must disclose on these points even when the disclosure is negative.

Sample size	Based on prior publications in our laboratory, we used n = 40-50 animals per genotype per lifespan analysis. For all other experiments, a minimum of n = 3 biologic replicates are used and specified in figure legends.
Data exclusions	Single outliers in a dataset were evaluated by Grubb's test. If an outlier was found ($p < 0.05$), it was removed and the remaining data are presented.
Replication	All experiments were conducted at least two independent times with successful replication. All experiments contain at least n = 3 biologic replicates in the final datasets.
Randomization	Animals were used from multiple litters at random, and control WT littermates were used.
Blinding	Most experiments were unblinded as animal genotype was carefully noted at each step.

Reporting for specific materials, systems and methods

We require information from authors about some types of materials, experimental systems and methods used in many studies. Here, indicate whether each material, system or method listed is relevant to your study. If you are not sure if a list item applies to your research, read the appropriate section before selecting a response.

Materials & experimental systems

n/a	Involved in the study	
☐	☒	Antibodies
☒	☐	Eukaryotic cell lines
☒	☐	Palaeontology and archaeology
☐	☒	Animals and other organisms
☒	☐	Human research participants
☒	☐	Clinical data
☒	☐	Dual use research of concern

Methods

n/a	Involved in the study	
☒	☐	ChIP-seq
☒	☐	Flow cytometry
☒	☐	MRI-based neuroimaging

Antibodies

Antibodies used	Primary antibodies used are: anti-p16 (1:1000; Abcam; ab189034), anti-ATG9A (1:1000; Abcam, clone EPR2450(2); ab108338), anti-FAM134B (1:4000; Abcam; ab151755), anti-Sec62 (1:1000; Abcam; ab140644), anti-pTau pSer396 (1:1000; Cell Signaling; 9632S), anti-Tau T46 (1:2000; ThermoFisher; 13-6400), anti-β-actin (1:1000; Cell Signaling Technology; 4967), anti-acetylated lysine (1:50; Cell Signaling Technology; 9441), anti-ATG9A (1:50; Abcam, clone EPR2450(2); ab108338), anti-Beta Amyloid (clone 6E10, 1:100, Signet), anti-NeuN (EMD Millipore; #ABN91MI; 1:1,000), anti-synaptophysin (abcam; #ab32127; 1:200), and anti-PSD95 (Thermo Fisher; #MA1-045; 1:200). Secondary antibodies used for visualization include: goat anti-rabbit or anti-mouse Alexa Fluor® 680-conjugated or Alexa Fluor® 800-conjugated secondary antibodies, and mouse anti-rabbit TrueBlot HRP-conjugated secondary antibody (Rockland; #18-8816-31).
Validation	Antibody validation was not conducted by the investigators but rather relied upon from prior publications or the antibody supplier.

Animals and other organisms

Policy information about studies involving animals; ARRIVE guidelines recommended for reporting animal research

Laboratory animals	Mouse, C57BL/6. The genotype, sex, and age varied for given experiments are specified in figure legends.
Wild animals	This study did not involve wild animals.
Field-collected samples	This study did not involve samples collected from the field.

Ethics oversight	All animal experiments were carried out in accordance with the National Institute of Health Guide for the Care and Use of Laboratory Animals and were approved by the Institutional Animal Care and Use Committee of the University of Wisconsin-Madison (protocol #M005120). Human fibroblasts were obtained from the University of Wisconsin-Madison Human Stem Cell Core and from Coriell with approval from the University of Wisconsin Human Subject IRB (protocol # 2014-0613). Research conducted with stored human tissue and cells does not constitute "clinical research" and falls under "Exemption 4" under HHS regulations at 45 CFR Part 46.

Note that full information on the approval of the study protocol must also be provided in the manuscript.
